# Bioactive phytochemicals in an aqueous extract of the leaves of *Talinum triangulare*

**DOI:** 10.1002/fsn3.449

**Published:** 2016-12-02

**Authors:** Catherine C. Ikewuchi, Jude C. Ikewuchi, Mercy O. Ifeanacho

**Affiliations:** ^1^ Department of BiochemistryFaculty of Science, University of Port HarcourtChobaNigeria

**Keywords:** benzoic acid derivatives, carotenoids, flavonoids, hydroxycinnamates, *Talinum triangulare*

## Abstract

An aqueous leaf extract of *Talinum triangulare* was screened for the presence of bioactive molecules, using gas chromatography coupled with pulse and flame ionization detectors. It had high carotenoids; moderate benzoic acid derivatives, hydroxycinnamates and flavonoids; and low terpenes, alkaloids, phytosterols, allicins, glycosides, saponins, and lignans contents. Ten known carotenoids (mainly 50.42% carotene and 33.30% lycopene), nine benzoic acid derivatives (mainly 84.63% ferulic acid and 11.92% vanillic acid), and six hydroxycinnamates (55.44% p‐coumaric acid and 44.46% caffeic acid) were detected. Also detected were eight lignans (88.02% retusin) and thirty flavonoids (50.35% quercetin and 39.36% kaempferol). The medicinal properties of the major components of these phytochemical families that were detected in the aqueous extract of the leaves were discussed herein and proposed to be explored for their potential health benefits. The great number of potentially active biomolecules and their multifunctional properties make *Talinum triangulare* a ready source of health‐promoting substances.

## 1 Introduction

*Talinum triangulare* (Jacq.) Willd. (Family: Portulaceae), is commonly called waterleaf. It is an herbaceous, annual, coalescent, and glabrous plant widely grown in tropical regions as a leafy vegetable. In Nigeria, it is consumed as a leafy vegetable and constituent of sauces (or vegetable soups). Nutritionally, it is a good source of some minerals (e.g., calcium, magnesium, and potassium) and vitamins (e.g., ascorbic acid and pyridoxine) (Oguntona, 1998). The extract from the leaves and roots is used to cure asthma (Ogie‐Odia & Oluowo, 2009). According to Ofusori et al. (2008), “*waterleaf consumption has benefiting effects on the neurons of the cerebrum and may probably enhance the cognitive ability in Swiss albino mice*”. In Edo State, Nigeria, *Talinum triangulare* is used as a diuretic, and for the management of gastrointestinal disorders (Mensah, Okoli, Ohaju‐Obodo, & Eifediyi, 2008). It is also used to treat *Shistosomiasis*, scabies, fresh cuts, high blood pressure, and anemia (Ogunlesi et al., 2010).

Preliminary phytochemical studies reported the presence of carotenoids (Ogbonnaya & Chinedum, 2013), alkaloids, flavonoids, saponins, and tannins in the leaves (Aja, Okaka, Onu, Ibiam, & Urako, 2010; Ukpabi, Akubugwo, Agbafor, Wogu, & Chukwu, 2013) and leaf extract (Swarna & Ravindhran, 2013) of *Talinum triangulare*. All these studies reported the total quantities of these families of compounds, without elucidating the individual compounds that constitute them. An attempt to identify these individual components by de Oliveira Amorim et al. (2014), yielded campesterol, sitosterol, stigmasterol, scotenol, 3‐(N‐acryloyl, and N‐pentadecanoyl) propanoic acid, allantoin, 3‐O‐bD‐glucopyranosyl‐sitosterol, 3‐O‐bD‐glucopyranosyl‐stigmasterol, (13^2^S,17R,18R)‐phaeophytin a**,** 17R,18R‐purpurin18 phytyl ester, ficuschlorin D acid, talichlorin A, 3^1^,3^2^‐didehydro‐15^1^‐hydroxyrhodochlorin‐15‐acetic acid d‐lactone‐15^2^‐methyl‐17^3^‐phytyl ester, and hydroperoxy‐ficuschlorin D. However, de Oliveira Amorim et al. (2014) did not quantify the detected compounds. To this end, this study profiled and quantified the phytochemical composition of an aqueous extract of the leaves of *Talinum triangulare*, and in addition discussed the bioactivities of the most abundant of the detected compounds, with a view to highlighting the possibilities of the use of the leaves as a functional food, or as a source of nutraceuticals.

## 2 Materials and Methods

### 2.1 Collection of plant samples and preparation of aqueous extract

Samples of fresh waterleaf plants were collected from within the Choba and Abuja Campuses of University of Port Harcourt, Nigeria. They were identified at the Herbarium of the Department of Plant Science and Biotechnology, University of Port Harcourt, Rivers State, Nigeria. They were then rid of dirt and their leaves were removed, oven dried at 55°C, and ground into powder. The powder was soaked in boiled distilled water for 12 hrs, after which the resultant mixture was filtered and the filtrate was evaporated to dryness. The percentage recovery of the crude extract was 2.296%. The residue obtained from the crude aqueous extract was subjected to phytochemical analysis.

### 2.2 General procedures

Gas chromatography was carried out at Multi‐environmental Management Consultants Limited, Igbe Road, Ikorodu, Lagos, with a Hewlett Packard HP 6890, gas chromatograph, fitted with HP Chemstation Rev. A09.01[1206] software, to identify and quantify the compounds. The standards were from Sigma‐Aldrich Co. and Lynnchem Biological Technology Co. Standard solutions were prepared in methanol for alkaloids, flavonoids, allicins and benzoic acid derivatives; acetone for carotenoids and lignans; methylene chloride for phytosterols and terpenes; and ethanol for hydroxycinnamates, glycosides and saponins. The linearity of the dependence of response on concentration was verified by regression analysis. Identification was based on comparison of retention times and spectral data with standards. Quantification was performed by establishing the calibration curves for each compound determined, using the standards.

### 2.3 Determination of phytochemical composition

The flavonoids’ extract was obtained in a similar way as was reported by Millogo‐Kone et al. (2009), and subjected to gas chromatography, with similar conditions as earlier reported by Ikewuchi, Onyeike, Uwakwe, and Ikewuchi (2011). The hydroxycinnamates’ extraction was carried out according to the method of Ortan, Popescu, Gaita, Dinu‐Pîrvu, and Câmpeanu (2009). The sample was extracted thrice with methanol, and the pooled extract was concentrated and subjected to gas chromatographic analysis, with similar conditions as earlier reported by Ikewuchi, Ikewuchi, and Ifeanacho (2015). The lignans’ extract was prepared according to the method of Chapman, Knoy, Kindscher, Brown, and Niemann (2006), and subjected to gas chromatography with similar conditions as earlier reported by Ikewuchi, Ikewuchi, and Ifeanacho (2014). The benzoic acid derivatives’ extract was prepared according to the method of Ndoumou, Ndzomo, and Djocgoue (1996), before being subjected to gas chromatography, with similar conditions as earlier reported by Ikewuchi et al. (2014).

The alkaloids’ extract was prepared according to the method of Tram, Mitova, Bankova, Handjieva, and Popov (2002), and subjected to gas chromatographic analysis, with conditions as earlier reported by Ikewuchi et al. (2014). The carotenoids’ extract was prepared according to the method of Rodriguez‐Amaya and Kimura (2004) by successively extracting acetone and a (1:1) mixture of diethyl ether and petroleum ether, concentrating and redissolving in acetone before saponifying and reextracting with a (1:1) mixture of diethyl ether and petroleum ether. The extract was dried and redissolved in petroleum ether before being subjecting to gas chromatography with similar conditions as earlier reported by Ikewuchi et al. (2012).

Extraction of oil was carried out according to the AOAC method 999.02 (AOAC International, 2006), while the analysis of sterols was carried out according to the AOAC method 994.10 (AOAC International, 2006) with similar gas chromatographic conditions as was earlier reported by Ikewuchi et al. (2011). The glycosides’ extract was obtained in a similar manner as the one reported by Oluwaniyi and Ibiyemi (2007), and subjected to gas chromatography, with similar conditions as earlier reported by Ikewuchi, Ikewuchi, Ifeanacho, Igboh, and Ijeh (2013). The allicins’ extraction was carried out in a similar way as reported by Roy, Shakleya, Callery, and Thomas (2006); and the resultant extract was subjected to gas chromatography with similar conditions as was earlier reported by Ikewuchi et al. (2013). The saponins’ extraction was carried out in a similar manner as the one reported by Guo, Zhang, and Liu (2009). The extract obtained was subjected to gas chromatography with similar conditions as was earlier reported by Ikewuchi et al. (2013). The terpenes’ extraction was carried out in a similar way as reported by Ortan et al. (2009). The resultant extract was subjected to gas chromatography, with similar conditions as was earlier reported by Ikewuchi et al. (2013).

## 3 Results and Discussion

The detected flavonoids (Table 1) consisted mainly of quercetin (50.3%), kaempferol (39.4%), apigenin (5.4%), isorhamnetin (3.7%) and luteolin (1.0%). The benzoic acid derivatives fraction (Table 1) consists mainly of ferulic acid (84.6%), vanillic acid (11.9%), 4‐hydroxybenzoic acid (1.8%), and gallic acid (1.4%). The hydroxycinnamates fraction consisted mainly of p‐coumaric acid (55.4%) and caffeic acid (44.5%); the lignans fraction consisted mainly of retusin (88.0%), galgravin (5.943%), dehydroabietic acid (2.5%), and apigenin‐4΄,7‐dimethyl ether (2.2%). As shown in Table 2, the carotenoids fraction consisted mainly of carotene (50.4%), lycopene (33.3%), malvidin (11.5%), and asta‐xanthin (4.2%); while the phytosterols fraction consisted mainly of sitosterol (99.0%), and glycosides fraction consisted mainly of arbutin (99.9%). The saponins fraction consisted mainly of avenacin‐B1 (76.8%) and avenacin‐A1 (23.0%); while the allicins fraction consisted of diallyl thiosulphinate (89.7%), methylallyl thiosulphinate (9.1%), and allyl methyl thiosulphinate (1.2%). The alkaloids fraction (Table 3) consisted mainly of indicine‐N‐oxide (52.1%), ellipcine (8.1%), crinane‐3α‐ol (4.5%), augustamine (4.4%), 1β,2β‐epoxyambelline (3.8%), cinchonine (3.8%), 13‐α‐hydrorhombifoline (3.5%), dihydro‐oxo‐demethoxyhaemanthamine (3.0%), oxoassoamine (3.0%), caffeine (2.3%), augustifoline (2.1%), 9‐octadecinamide (2.0%), theobromine (2.0%), thalicarpin (1.7%), lupanine (1.4%), and crinamidine (1.3%). The terpenoids fraction (Table 4) consisted mainly of limonene (65.1%), camphor (5.0%), 1,8‐cineole (3.7%), terpinen‐4‐ol (2.9%), borneol acetate (2.4%), geranyl acetate (2.2%), neral (1.9%), borneol (1.6%), β‐pinene (1.5%), camphene (1.5%), sabinene (1.4%), neryl acetate (1.1%), citronellol (1.1%), and β‐amyrin (1.0%).

**Table 1:** Isolated and detected flavonoids, benzoic acid derivatives, hydroxycinnamates, and lignans in an aqueous extract of the leaves of *Talinum triangulare*

Compounds	Composition (mg kg^−1^)	Compounds	Composition (mg kg^−1^)
(+)‐Catechin	0.00017 ± 0.00013	**Benzoic acid derivatives**	
Resveratrol	0.000062 ± 0.000025	4‐Hydroxybenzaldehyde	0.00035 ± 0.00015
Genistein	0.000067 ± 0.000025	4‐Hydroxybenzoic acid	0.049 ± 0.044
Daidzein	0.000067 ± 0.000020	4‐Hydroxybenzoic acid methyl ester	0.0000065 ± 0.00000014
Apigenin	0.29 ± 0.10	Vanillic acid	0.33 ± 0.0061
Daidzin	0.000091 ± 0.000014	Gallic acid	0.037 ± 0.031
Butein	0.00013 ± 0.000043	Ferulic acid	2.3 ± 0.17
Naringenin	0.0039 ± 0.0012	Capsaicin	0.0050 ± 0.0049
Biochanin	0.00025 ± 0.000070	Rosmarinic acid	0.0028 ± 0.0012
Luteolin	0.054 ± 0.011	Tannic acid	0.0000083 ± 0.0000073
Kaempferol	2.1 ± 0.57	Total benzoic acid derivatives	2.7 ± 0.17
(−)‐Epicatechin	0.00043 ± 0.000083	**Hydroxycinnamates**	
(−)‐Epigallocatechin	0.000044 ± 0.0000032	p‐Coumarin	0.0013 ± 0.00047
Quercetin	2.7 ± 0.67	p‐Coumaric acid	1.2 ± 0.069
Gallocatechin	0.00024 ± 0.000097	Caffeic acid	0.98 ± 0.053
(−)‐Epicatechin‐3‐gallate	0.000030 ± 0.000002	Scopoletin	0.00019 ± 0.000062
(−)‐Epigallocatechin‐3‐gallate	0.000027 ± 0.0000029	Chlorogenic acid	0.00028 ± 0.00014
Isorhamnetin	0.20 ± 0.037	Chicoric acid	0.00029 ± 0.000013
Robinetin	0.000094 ± 0.0000032	Total hydroxycinnamates	2.2 ± 0.12
Ellagic acid	0.00015 ± 0.000049	**Lignans**	
Myricetin	0.000077 ± 0.000015	2‐Allyl‐5‐ethoxy‐4‐methoxyphenol	0.00020 ± 0.00018
Baicalein	0.00011 ± 0.000023	(9E, 12E, 15E)‐9, 12, 15‐Octadecatrien‐1‐ol	0.000044 ± 0.000037
Nobiletin	0.00014 ± 0.000032	Apigenin‐4΄,7‐dimethyl ether	0.00066 ± 0.00040
Baicalin	0.00011 ± 0.000027	Dehydroabietic acid	0.00073 ± 0.00047
Tangeratin	0.000042 ± 0.0000011	Retusin	0.026 ± 0.0043
Artemetin	0.000038 ± 0.000015	Galgravin	0.0018 ± 0.0016
Silymarin	0.000065 ± 0.0000017	Epieudesmin	0.000054 ± 0.000018
Naringin	0.000059 ± 0.000031	Sakuranin	0.000098 ± 0.000023
Rutin	0.000011 ± 0.0000039	Total lignans	0.030 ± 0.0066
Hesperidin	0.000014 ± 0.000014		
Total flavonoids	5.4 ± 1.4		

Values are mean ± SD (standard deviation) of duplicate determinations.

**Table 2:** Isolated and detected carotenoids, phytosterols, glycosides, saponins and allicins in an aqueous extract of the leaves of *Talinum triangulare*

Compounds	Composition (mg kg^−1^)
Carotenoids	
Malvidin	2.1 ± 0.16
β‐Cryptoxanthin	0.0070 ± 0.00051
Lycopene	6.12 ± 0.037
Carotene	9.3 ± 0.062
Lutein	0.0086 ± 0.00066
Xanthophyll	0.00083 ± 0.00013
Anthera‐xanthin	0.028 ± 0.022
Asta‐xanthin	0.78 ± 0.071
Viola‐xanthin	0.075 ± 0.0079
Neo‐xanthin	0.0013 ± 0.00013
Total carotenoids	18.0 ± 0.34
Phytosterols	
Cholesterol	0.0000078 ± 0.000000010
Cholestanol	0.00050 ± 0.000089
Ergosterol	0.00068 ± 0.00024
Campesterol	0.00094 ± 0.000015
Stigmasterol	0.0034 ± 0.0011
5‐Avenasterol	0.0034 ± 0.0011
Sitosterol	0.88 ± 0.13
Total phytosterols	0.89 ± 0.13
Glycosides	
Arbutin	0.093 ± 0.011
Salicin	0.000042 ± 0.000031
Amygdalin	0.000022 ± 0.0000032
Total glycosides	0.094 ± 0.011
Saponins	
Avenacin‐A1	0.012 ± 0.00038
Avenacin‐B1	0.039 ± 0.011
Avenacin‐A2	0.000029 ± 0.0000096
Avenacin‐B2	0.000043 ± 0.000026
Total saponins	0.051 ± 0.010
Allicins	
Diallyl thiosulphinate	0.020 ± 0.00027
Methylallyl thiosulphinate	0.0020 ± 0.00038
Allyl methyl thiosulphinate	0.00026 ± 0.0000089
Total allicins	0.022 ± 0.00066

Values are mean ± SD (standard deviation) of duplicate determinations.

**Table 3:** Isolated and detected alkaloid composition of an aqueous extract of the leaves of *Talinum triangulare*

Compounds	Composition (mg kg^−1^)
Choline	0.00046 ± 0.00033
Trigonelline	0.0012 ± 0.00079
Theobromine	0.0030 ± 0.00
Theophylline	0.00089 ± 0.00026
Caffeine	0.0035 ± 0.00
Augustifoline	0.0033 ± 0.00
Sparteine	0.0011 ± 0.00078
Ellipcine	0.012 ± 0.0085
Lupanine	0.0022 ± 0.0021
13‐α‐Hydrorhombifoline	0.0053 ± 0.0024
9‐Octadecinamide	0.0031 ± 0.00023
Dihydro‐oxo‐demethoxyhaemanthamine	0.0047 ± 0.0013
Augustamine	0.0067 ± 0.0045
Oxoassoamine	0.0047 ± 0.0023
Crinane‐3α‐ol	0.0070 ± 0.00093
Cinchonine	0.0058 ± 0.0048
Buphanidrine	0.0015 ± 0.00023
Cinchonidine	0.0013 ± 0.00053
Indicine‐N‐oxide	0.081 ± 0.017
Powelline	0.00060 ± 0.000012
Undulatine	0.00021 ± 0.0000094
Ambelline	0.000088 ± 0.0000095
6‐Hydroxybuphanidrine	0.000088 ± 0.000044
Acronycine	0.000069 ± 0.000018
Monocrotaline	0.000042 ± 0.00
6‐Hydroxypowelline	0.000068 ± 0.000028
Nitidine	0.00023 ± 0.00
Crinamidine	0.0020 ± 0.0019
6‐Hydroxyundulatine	0.00077 ± 0.00
1β,2β‐Epoxyambelline	0.0059 ± 0.00
Epoxy‐3,7‐dimethyoxycrinane‐11‐one	0.00068 ± 0.00053
Echitammidine	0.00061 ± 0.00
Akuammidine	0.0015 ± 0.00
Voacangine	0.00026 ± 0.00
Mitraphylin	0.00046 ± 0.00
Camptothecin	0.00022 ± 0.00
Echitamine	0.00027 ± 0.00
Colchicine	0.00028 ± 0.00
Tetrandrine	0.00021 ± 0.00
Emetine	0.00050 ± 0.00
Thalicarpin	0.0026 ± 0.00
Paclitaxel	0.000093 ± 0.00
Total alkaloids	0.15 ± 0.00063

Values are mean ± SD (standard deviation) of duplicate determinations.

**Table 4:** Isolated and detected terpene composition of an aqueous extract of the leaves of *Talinum triangulare*

Compounds	Composition (mg kg^−1^)
α‐Pinene	0.0013 ± 0.0000011
β‐Pinene	0.0022 ± 0.0000096
Limonene	0.093 ± 0.0068
Cis‐ocimene	0.00029 ± 0.00000017
Myrcene	0.00064 ± 0.00024
Alloocimene	0.0010 ± 0.00000015
Camphene	0.0022 ± 0.0012
Sabinene	0.0020 ± 0.00059
α‐Thujene	0.0014 ± 0.00022
Camphor	0.0072 ± 0.0037
Neral	0.0027 ± 0.00026
1,8‐Cineole	0.0053 ± 0.0026
Borneol	0.0023 ± 0.00013
Nerol (geraniol)	0.00083 ± 0.0000000035
α‐Terpineol	0.0011 ± 0.0000000020
Terpinen‐4‐ol	0.0042 ± 0.0014
Citronellol	0.0015 ± 0.0000000010
Borneol acetate	0.0035 ± 0.0012
Neryl acetate	0.0016 ± 0.00028
Geranyl acetate	0.0031 ± 0.0011
Taraxeron	0.0014 ± 0.00000021
α‐Amyrin	0.0013 ± 0.00000021
β‐Amyrin	0.0014 ± 0.000060
Lupeol	0.0014 ± 0.000000013
Total terpenoids	0.14 ± 0.016

Values are mean ± SD (standard deviation) of duplicate determinations.

The results showed the presence of bioactive compounds in the aqueous extract of the leaves of *Talinum triangulare*. These compounds have a wide range of biological properties. For example, quercetin has analgesic, antiallergenic, antibacterial, antidiabetic, anti‐inflammatory, and antiviral activities (Prabha, Dahms, & Malliga, 2014). Studies have shown that kaempferol has a wide range of biological activities, including antioxidant, anti‐inflammatory, antimicrobial, anticancer, cardio‐protective, neuroprotective, antidiabetic, antiosteoporotic, estrogenic/antiestrogenic, anxiolytic, hepatoprotective, analgesic, and antiallergic activities (Calderón‐Montaño, Burgos‐Morón, Pérez‐Guerrero, & López‐Lázaro, 2011). Numerous studies have demonstrated the antioxidant, antihypertensive, anticancer, and antiosteoporotic properties of lycopene, as well as its ability to protect against cardiovascular diseases, and amyotrophic lateral sclerosis (Rao & Rao, 2007). β‐Carotene is an antioxidant and antiosteoporotic agent (Rao & Rao, 2007), and a major precursor of vitamin A (Agea et al., 2014). Malvidin possesses antioxidant, anti‐inflammatory, cardio‐protective, antihypertensive, and antitumor properties (Huang, Zhu, Li, Sui, & Min, 2016; Quintieri et al., 2013; Seo et al., 2016). Therefore, a great number of potentially active molecules present in the leaves of *Talinum triangulare*, as well as the multifunctional properties these compounds, make *Talinum triangulare* a good source of health‐promoting substances.

## Conflict of interest

We declare that we have no conflict of interest.
